# La Grippe

**Published:** 1890-03

**Authors:** H. B. Stiles

**Affiliations:** Abilene, Texas


### LA GRIPPE.

H. B. Stiles, A. M., M. D., Abilene, Texas.

Since writing my last report upon la grippe (Homœopathic Physician, February No., page 86), I have had a little more experience with the disease, and can report a most speedy and homœopathic remedy for the tormenting frontal (and other) neuralgia which in this country is an almost certain sequel of the disease.

In Dr. Jno. Butler's text-book of Electro-Therapeutics, p. 31, in the pathogenesis of electricity, will be found the following symptom: "Pain tingling, acute, darting, lancinating * * * sometimes producing great agony." These are the indications for this mighty polychrest. Belladona, Spigelia, and Chin-ars. do relieve this neuralgia, but not with the certainty and celerity of the Faradic current.

In numerous cases Dr. Evarts and myself have used our portable Faradic battery with results most gratifying to our patients and ourselves.

Owing to the extreme sensitiveness of the affected parts, we must make the current very light. So we place a rheostat in circuit by which we can graduate the current to any attenuation. Stroking the part (usually forehead) with the electrodes for five or ten minutes usually relieves the pain completely. In some cases we have to repeat the process in half an hour, and in severe cases it is necessary to treat the patient on two or three days. But ordinarily the action of the current is quicker than that of any other homœopathic remedy—though, of course, it must be clearly indicated. It is vastly superior to the stupefying morphine with which the "scientifics" have rendered their confiding friends oblivious to pleasure and pain. It relieves without stupefying.

A very small battery is sufficient—one that can be obtained for five dollars, and even that is likely to be too strong, necessitating the interposition of the rheostat.

Our static and galvanic batteries also relieve cases that come to the office, but being non-portable we do not use them so frequently as the Faradic.

Another Case.—Sam S., æt. ten. I was called in haste to see this boy, who had suffered three or four days with la grippe before I was called.

His mother reported that two hours before I came he was seized with a spasm which is briefly described as a severe opisthotonus. She said his head and heels came within fifteen inches of each other, while every joint in his body seemed to crack. The symptoms were: red face, moderate fever, dry mouth, extreme nervousness, starting and jumping in sleep, inclined to stupor. ℞. Belladonna, at eight P. M.

At two A. M. his father called me. Reported child as worse. Had no more spasms, but seemed to be approaching them. More nervous; boring head into pillow, gritting teeth, face flushed on one side, pain in epigastrium. ℞. Chamomilla, sending it by the father. Followed myself in half an hour. Found the medicine had acted already and somewhat soothed the nervousness. So I continued it, though I confess that appearances then were more suggestive of Opium. But I remembered Dr. G. J. Jones' advice to "let well enough alone" when medicine is acting properly. The child continued to improve, receiving a dose whenever he seemed worse, which was about once an hour during the rest of the night. In two days he was practically well.

---

